# Correction to “Telemedicine in epilepsy care: A Nationwide survey by the Italian Chapter of the International League Against Epilepsy (LICE) on adoption, barriers, and perceived value”

**DOI:** 10.1002/epi4.70307

**Published:** 2026-07-18

Brigo F , Assenza G , Dono F , Duca M , Lanzone J , Lo Barco T , et al. Telemedicine in epilepsy care: A Nationwide survey by the Italian Chapter of the International League Against Epilepsy (LICE) on adoption, barriers, and perceived value. Epilepsia Open. 2026;11:516–526. 10.1002/epi4.70220 41618811 PMC13051808

The institutional affiliation for one of the authors, Simona Lattanzi, was incorrectly stated as “Epilepsy Center, C. Poma Hospital, Mantua, Italy.” The correct affiliation for this author is “Neurological Clinic, Department of Experimental and Clinical Medicine, Marche Polytechnic University, Ancona, Italy.”

We apologize for this error.

